# Perioperative Considerations, Anesthetic Management and Transesophageal Echocardiographic Evaluation of Patients Undergoing the Ross Procedure

**DOI:** 10.3390/jcdd12040126

**Published:** 2025-03-31

**Authors:** Giacomo Scorsese, Brandon Yonel, Eric Schmalzried, Alexandra Solowinska, Zhaosheng Jin, Jeremy Poppers

**Affiliations:** 1 Department of Anesthesiology, Stony Brook University Hospital, Stony Brook, NY 11794, USA; brandon.yonel@stonybrookmedicine.edu (B.Y.); eric.schmalzried@stonybrookmedicine.edu (E.S.); jeremy.poppers@stonybrookmedicine.edu (J.P.); 2 Renaissance School of Medicine, Stony Brook University, Stony Brook, NY 11794, USA; alexandra.solowinska@stonybrookmedicine.edu

**Keywords:** Ross procedure, valvular heart disease, aortic valve, transesophageal echocardiography, cardiopulmonary bypass, autologous normovolemic hemodilution

## Abstract

The Ross procedure introduced a new technique for aortic valve replacement by utilizing a pulmonary autograft to replace the diseased aortic valve. This approach provides a living, dynamic valve substitute capable of growth and adaptation to systemic pressures while addressing the limitations of mechanical valves, which require lifelong anticoagulation, and bioprosthetic valves, which lack durability and growth potential. The Ross procedure offers superior hemodynamic performance and freedom from anticoagulation. While initially popular, utilization declined due to its technical complexity and concerns regarding the potential for the failure of two valves, requiring additional operations. Advances in surgical techniques, such as reinforced autografts, improved myocardial protection, and better homograft preservation, coupled with evidence of favorable long-term outcomes, have renewed interest in the procedure. Preoperative imaging with echocardiography, cardiac magnetic resonance imaging, and computed tomography angiography ensures optimal patient selection and preparation. Intraoperatively, precise autograft harvesting, accurate implantation, and meticulous right ventricular outflow tract reconstruction are critical for success. Blood conservation techniques, such as acute normovolemic hemodilution and retrograde autologous priming, are employed to minimize transfusion-related complications. The anesthesiologist plays a critical role, including meticulous monitoring of myocardial function and hemodynamics, with intraoperative transesophageal echocardiography being essential for assessing valve integrity and ventricular function. Recent studies suggest that the Ross procedure can restore life expectancy in appropriately selected patients, reinforcing its value as a surgical option for managing aortic valve disease.

## 1. Introduction

The Ross procedure, first described by British cardiac surgeon Donald Ross in 1967, is a landmark surgical technique that introduced the use of a pulmonary autograft for aortic valve replacement [1]. Based on previous experimental work in canine models [2], this technique aimed to address some of the limitations of existing valve replacement options, particularly in younger and more active patients. By replacing the diseased aortic valve with the patient’s pulmonary valve and reconstructing the right ventricular outflow tract with an allograft, the Ross procedure offered a dynamic, living valve replacement that could potentially grow along with pediatric patients and effectively adapt to the higher-pressure systemic circulation [3]. Over 50 years later, the Ross procedure continues to be an excellent option for select patient populations [4].

Around 85,000 aortic valve replacements are conducted annually in the United States, making it the most frequently performed type of valve replacement [5]. The first mechanical valves were introduced in 1952 [6], while bioprosthetic valves came slightly later. Mechanical valves such as the Starr–Edwards ball-in-cage mechanical valve, introduced at a conference in 1960 [7], provided exceptional durability. However, due to the significant thromboembolic risks imposed by mechanical valves, patients would require lifelong anticoagulation therapy, subsequently increasing the risk of bleeding complications [8]. Valve replacement with a bioprosthetic valve was first performed with an allograft (also referred to as a homograft) in 1962 [9], which eliminated the requirement for anticoagulation and offered improved hemodynamic qualities compared to mechanical valves. However, allografts are rarely used in clinical practice due mainly to limited availability. Xenografts, derived from porcine or bovine tissues, are more widely available but share similar limitations to allograft valves as both types are hampered by variable durability and lack of growth potential [10,11].

It was within this context that the Ross procedure was proposed. It was hypothesized that structural similarities between the pulmonary and aortic valves could allow the pulmonary valve to serve as an ideal replacement for the aortic valve [12]. The pulmonary autograft could withstand systemic pressures when transplanted to the aortic position, and the right ventricular outflow tract could be reconstructed with a homograft, which would function adequately in the low-pressure pulmonary circulation [13]. This novel approach offered the potential for a living, autologous tissue replacement with superior hemodynamic performance and durability without the burden of anticoagulation [14].

The Ross procedure gained popularity as a promising option for patients with aortic valve disease, particularly younger individuals seeking long-term solutions that avoid lifestyle or activity limitations [14]. However, utilization peaked in the late 1990s [15], representing <0.1% of all aortic valve replacements by 2010 [16]. Several factors contributed to this decline, including the technical complexity of the operation, the potential for failure of two valves (the pulmonary autograft and the homograft), and the risks associated with reoperations [17]. Nevertheless, persistent efforts by high-volume centers and ongoing research into the long-term outcomes of the Ross procedure have led to a resurgence of interest in recent years due to accumulating evidence of its favorable long-term outcomes [18,19], with estimated 25-year survival rates, based on retrospective analysis of over 2000 patients, without significant difference to that of the general population in appropriately selected patients [20,21,22]. Advances in surgical techniques, such as reinforced autografts, improved myocardial protection, better homograft preservation, and proactive management of the ascending aorta, have further improved outcomes [23,24,25,26].

Currently, the Ross procedure is primarily recommended for young and middle-aged adults with aortic valve disease who either have contraindications to anticoagulation or wish to avoid long-term anticoagulation therapy [27]. While a set of definitive indications exists, we summarize them as any patient with aortic valve disease (i.e., aortic stenosis (AS) or aortic insufficiency (AI)) and life expectancy greater than 15–20 years who also desire freedom from lifelong anticoagulation and engage in high-impact physical activity [4]. The ideal candidates are patients with isolated aortic valve disease, particularly those with aortic stenosis, who do not have a dilated aortic annulus, significant connective tissue disorders, or other contraindications that could compromise the integrity of the pulmonary autograft [28]. As such, the patient population routinely encountered in the adult cardiac operating room are those with bicuspid aortic valve (BAV) and concomitant AS, AI, and/or ascending aortic aneurysm—characteristically young (<70 years old) and predominantly male.

The explosive growth in the management of aortic valve disease has ushered in a wide range of both surgical and transcatheter-based techniques. However, the Ross procedure offers the only viable, living valve substitute for the diseased aortic valve [16]. Ongoing research into the long-term durability of the procedure and the development of less invasive techniques may expand its applicability in the future [29]. While this surgery has gone through numerous iterations and fallen into and out of favor, there seems to be a paucity of literature regarding its anesthetic management. As such, the authors’ current understanding is adapted from isolated reports and institutional experiences in adult cardiac surgery. This review aims to provide a brief overview of the Ross procedure, including technical considerations, its contemporary relevance in managing aortic valve disease, as well as anesthetic management.

## 2. Anatomical Considerations

The aortic valve (AV) is a three-dimensional structure with three semilunar leaflets, known as the right, left, and non-coronary cusps, that are suspended within the functional aortic annulus—a ring of connective tissue. The right coronary ostia and left main coronary ostia originate from their respective left coronary cusps (LCCs) and right coronary cusps (RCCs). The RCCs and right coronary ostia are the most anterior structures. These structures are intrinsically joined and disease of just one component can lead to abnormalities of the others over time [30]. This delicate apparatus shares a corresponding counterpart situated orthogonal to it, known as the pulmonic valve (PV).

The PV is anterior to the native AV and originated from the same primordial structures in the embryological human heart. The PV divides the right ventricular outflow tract (RVOT) and pulmonary trunk through three semilunar cusps: the left, right, and anterior. The subvalvular apparatus separates itself from adjacent structures with a muscular fold known broadly as the infundibulum, a key structure utilized not only for naming ventricular septal defects but also in the Ross procedure to reconstruct the neo-LVOT [31].

Valvular disease in either of these can be distilled into two generalized pathologies: stenosis, in which there is a narrowing of the valve orifice, which limits systolic antegrade flow through the valve, or regurgitation, comprising retrograde diastolic flow through the valve [32]. The etiology of these two disease states can take on a multiplicity of etiologies, which range from congenital structural heart abnormalities, infective endocarditis, senile calcific plaques, rheumatic disease, and genetic connective tissue disorders. Furthermore, pathologies can present in a mixed picture of combined stenosis and regurgitation. At any rate, while symptomatology may develop over many years, their presence typically does not appear until the condition is severe, at which point morbidity and mortality are very high [32].

## 3. Preoperative Assessment

Accurate and thorough preoperative evaluation is essential in all cardiac surgical procedures to optimize patient safety and guide perioperative planning. For the Ross procedure, additional imaging and anatomic assessment are required to determine candidacy due to the complexity and autologous nature of the operation. Accordingly, the preoperative evaluation is divided into two subsections: routine preoperative investigations conducted for all cardiac surgeries and Ross-specific investigations focused on determining the feasibility of autograft transplantation.

### 3.1. Routine Preoperative Investigations

These investigations are performed for most patients undergoing major cardiothoracic surgery and establish a baseline profile to identify modifiable risk factors, optimize perioperative management, and guide surgical and anesthesia planning. Although additional laboratory studies may be obtained based on patient factors, the following laboratory studies are obtained for all patients:

- A complete blood count (CBC) is used to evaluate for anemia, infection, and platelet abnormalities, providing baseline hematologic status.
- A comprehensive metabolic panel (CMP) assesses renal function, glucose, and electrolyte balance to optimize perioperative management.
- Coagulation studies, including prothrombin time (PT) and activated partial thromboplastin time (aPTT), screen for bleeding or clotting risks that influence intraoperative anticoagulation and transfusion planning.
- Blood typing and crossmatching ensure that compatible blood products are available should transfusion be required.

To further determine the patient’s baseline hemodynamic profile and provide critical information to guide perioperative and intraoperative management strategies, the following imaging studies are routinely obtained:

- Electrocardiography (ECG) identifies arrhythmias, conduction abnormalities, or ischemic changes that may affect anesthetic and surgical risk.
- A chest radiograph (CXR) is obtained to evaluate the cardiac silhouette and pulmonary parenchyma and to identify any occult thoracic pathology.
- Transthoracic echocardiography (TTE) provides an initial noninvasive assessment of cardiac structure and function, including valvular morphology, ventricular performance, and chamber dimensions.

Furthermore, in patients with additional cardiovascular risk factors, bilateral carotid duplex and coronary angiography or coronary computed tomography angiography (CTCA) would be performed to detect obstructive coronary disease that may require simultaneous revascularization [16].

### 3.2. Ross Procedure-Specific Investigations

Patients being considered for the Ross procedure require a series of detailed imaging evaluations aimed at assessing anatomic and structural compatibility, functional requirements, and surgical feasibility for pulmonary autograft transplantation [33].

- Transthoracic echocardiography (TTE) continues to play a central role, offering initial information on the structure and function of both the aortic and pulmonary valves, as well as left ventricular systolic performance. Doppler imaging augments this evaluation by quantifying flow velocities and valvular properties [29].
- Although uncommon and avoided, if possible, transesophageal echocardiography (TEE) is an invasive modality that offers superior sonographic windows and spatial resolution, leading to more precise visualization of the aortic root, pulmonary valve, and ascending aorta in the case of suboptimal TTE.
- Computed tomography coronary angiography (CTCA) is used to scrutinize coronary artery architecture and identify variations such as a short left main or anomalous origin of the right coronary artery, which may complicate autograft harvesting and reimplantation [16].
- Further anatomic and functional detail is provided by cardiac magnetic resonance imaging (cMRI), which provides more advanced anatomical and functional insights and is particularly useful for characterizing valvular flow dynamics, ventricular function, perfusion, and anomalous coronary arteries [34].
- Cardiac computed tomography angiography (CTA) offers high-resolution cross-sectional imaging, which complements cMRI [29]. CTA is employed to examine the aortic root and ascending aorta, particularly in cases where dilation is suspected. Measurements exceeding 38–40 mm typically prompt consideration of concomitant surgical intervention [19].

A focused evaluation of the pulmonary valve complex is required to assess its suitability for transfer to the aortic position. This includes analysis of leaflet morphology, annular size, tissue integrity, and infundibular musculature. Variations of the main pulmonary artery and pulmonary valve (PV) complex—such as the presence of a quadricuspid or bicuspid PV—may preclude its use as an autograft [35]. The infundibulum is assessed for muscle thickness, strength, and relationship to surrounding structures, as these factors influence autograft harvesting and RVOT reconstruction.

The aforementioned characteristics are also used to determine the compatibility of the pulmonary root complex with the aortic annulus. In addition, the relationship of the pulmonary root to nearby structures, including the left main coronary artery and left atrial appendage, is carefully examined to avoid intraoperative injury during harvesting [16].

## 4. Intraoperative Technical Considerations

### 4.1. Cannulation Strategies

Aortic cannulation is typically achieved through the aortic arch, beyond the takeoff of the innominate artery using a Seldinger technique to maximize the length of the working aorta by cross-clamping just proximal to the innominate artery [36,37]. This approach requires careful coordination with the anesthesiology team to correctly identify the surgeon’s wire in the lumen of the proximal descending aorta, using transesophageal echocardiography (TEE). The benefits of the arch cannulation strategy are that the aortic cross-clamp may be placed distal to the PA bifurcation, which facilitates autograft harvesting and distal pulmonary autograft anastomosis [36,37]

Venous cannulation is routinely performed through a bi-caval approach, in which a single-stage cannula is placed in the superior and inferior vena cava, respectively. Right atrial cannulation using a multistage cannula has also been utilized in some cases; a choice influenced by factors such as the presence of a patent foramen ovale (PFO) or the need for collateral intracardiac procedures [38].

Left ventricular (LV) venting is an important adjunct of myocardial protection during the Ross procedure, which is performed before the successful delivery of cardioplegia. Conventionally, the LV vent is placed via the right superior pulmonary vein and directed toward the LV through the mitral valve. The LV vent is inserted by the surgical team and its position is confirmed by TEE. Attention should be paid to ascertain that the tip of the LV vent does not impinge on the mitral or aortic valve [39].

Adequate myocardial protection is paramount given the prolonged aortic cross-clamp times frequently required for the Ross procedure and the potential need for ascending aortic repair vs. replacement. A combination of antegrade and retrograde cardioplegia ensures optimal distribution, especially in the presence of coronary artery disease or severe ventricular hypertrophy [40]. When retrograde cardioplegia is utilized, the position of the coronary sinus catheter is confirmed via TEE guidance to minimize the risk of injury prior to injection. Del Nido cardioplegia is usually utilized to arrest the heart; however, the choice of cardioplegia can be surgeon-dependent. Systemic hypothermia (20–25 °C) provides additional myocardial protection and facilitates a safe period of circulatory arrest to minimize ischemic injury [4].

### 4.2. Autograft Harvesting and Implantation

Pulmonary autograft harvesting is critical to prevent structural damage and improve long-term durability. The pulmonary root is explanted en bloc, with particular care taken to preserve valvular integrity and the delicate infundibular muscle cuff. Excess fat and connective tissue are trimmed, allowing for a tension-free anastomosis. The autograft is then transferred to the aortic position, where it can be implanted using either the sub-coronary or total root replacement technique [40]; a decision driven primarily by surgeon preference.

The sub-coronary implantation technique involves excising the patient’s native aortic valve and suturing the autograft in a scalloped fashion below the coronary ostia. While there is a long-standing debate amongst operators over the durability of the sub-coronary versus total root technique, the former is less commonly used due to its technical complexity and the potential for distortion of the autograft’s original shape, which limits its use in patients with aortic root asymmetry (i.e., Sievers Type 0, bicuspid AV, unicuspid AV, and aortic root aneurysms) [37]. The total root replacement technique, in contrast, involves excising the native aortic root and replacing it entirely with the pulmonary autograft. This is the primary approach used at our institution since it allows the positioning of the autograft to be more anatomic, thus reducing the risk of geometric distortion [4]. Regardless of the implantation technique, accurate autograft sizing and symmetrical commissural alignment both decrease the incidence of future valvular insufficiency.

### 4.3. Right Ventricular Outflow Tract Reconstruction

The final step in the Ross procedure is the reconstruction of the RVOT, most commonly using a pulmonary homograft, which accounted for over 83% of Ross procedure pulmonary valve conduits in the United States in 2022 [41]. Several options exist for homograft preservation, including non-decellularized cryopreservation, decellularization, and fresh antibiotic sterilization.

Cryopreserved pulmonary homografts (cadaveric human pulmonary valve and artery) have been historically used, and remain the preferred conduit at our institution and in the United States overall. This can be attributed to the cryopreserved pulmonary homograft’s track record of reliable performance, favorable hemodynamics, and avoidance of anticoagulation, especially in adults and older children [42]. Cryopreserved homografts are however known to elicit a host immune-mediated inflammatory reaction, thus leading to homograft dysfunction (i.e., stenosis). Decellularized homografts, in which the donor cells are removed through various chemical treatments, have gained some popularity due to their potential for reducing immunogenicity and improving long-term preservation of valve integrity [43]. Regardless of the choice, the homograft is trimmed to an appropriate length and anastomosed to the right ventricular infundibulum proximally and the pulmonary artery distally [44].

Notably, patients are typically started on 6 months of nonsteroidal anti-inflammatory drugs (NSAIDs) postoperatively in order to decrease this inflammatory response [37,44].

## 5. Blood Conservation Techniques

In cardiac surgery, postoperative bleeding is one of the most germane complications and, within a national blood supply range, it accounts for 15% to 20% of total transfusion requests [45]. This need originates from the nature of the unique interaction between the cardiopulmonary bypass circuit and clotting elements within the patient’s blood.

Platelets express a range of surface molecules that mediate their hemostatic and inflammatory functions. The CPB circuit, comprised of polyvinylchloride (PVC) tubing, activates platelets with resultant structural and biochemical changes. Alterations in platelet count, clot formation, and the peak platelet force development after CPB are all substantially lower than before CPB, subsequently playing a role in CPB-associated microvascular hemorrhage [46,47]. Moreover, despite supratherapeutic heparin anticoagulation, some activation of coagulation still occurs and increases with the duration of CPB. The combination of contact activation on foreign surfaces within the bypass circuit, exposure of blood to air and Tissue Factor in the wound, and recirculation of this blood via cardiotomy suction, results in the depletion of key clotting factors [48].

### 5.1. Acute Normovolemic Hemodilution

Acute normovolemic hemodilution (ANH) is a blood conservation technique first described in the 1970s to decrease the need for intraoperative allogenic blood transfusion. The procedure is accomplished by removing a portion of the patient’s blood preoperatively, which is stored and later re-infused. The relevance of this technique in surgical literature outside the cardiac operating room has been debated, and current evidence supporting ANH lacks strength; however, its physiological relevance in cardiopulmonary bypass (CPB) cannot be understated. Further, the efficacy of ANH use for cardiac surgery may soon be clarified by a multicenter randomized controlled trial currently in progress [49].

One of the key concepts in applying hemodilution is to define a patient’s “safe” lower limit for hematocrit. Patients typically have a considerable reserve of red cells, which is the principal reason that the acute removal of blood in the preoperative period is a viable therapeutic option [50]. Although ANH is selectively employed as part of our institution’s Ross protocol, we generally do not utilize the technique for routine cardiac surgeries. Hemodilution from the CPB priming fluid represents a clinical concern in the safe implementation of ANH, especially in patients with known cardiovascular disease. Critical red cell mass is the lower limit of hemoglobin associated with effective oxygen delivery. At hematocrits less than 20–25%, myocardial metabolism may be compromised by the decreased supply and heightened oxygen demand [50,51].

While patient and procedure variations ultimately dictate clinical decision-making, we generally isolate ANH to patients with a starting HCT of at least 35%. Additionally, we utilize the Maximal Allowable Blood Loss equation to ensure a safe volume of blood is being removed while remaining above a lower HCT limit of 30% in lieu of the obligatory hemodilution imposed during the initiation of CPB. After central line insertion, 2 units (450 cc each) of blood are sterilely drawn from the central line into a Citrate–Phosphate–Dextrose–Adenine (CPDA) collection bag. Harvest volume is measured using a blood rocker and scale to ensure the correct ratio of blood to citrate anticoagulant in each CPDA bag is not exceeded. After removal, an equivalent amount of crystalloid (Ringer’s lactate at our institution) or colloid is infused into the patient. The blood is then labeled, covered with a towel, and stored on a rocker at room temperature (to maintain platelet function), until being re-infused following separation from CPB and completion of protamine reversal (typically within 6 h).

### 5.2. Retrograde Autologous Priming

The initiation of cardiopulmonary bypass (CPB) comes with unique challenges and downstream consequences, namely hemodilution. Generally speaking, a typical CPB circuit requires a priming volume of approximately 1500 mL of crystalloid solution [52]. This volume is ultimately infused into the patient with the initiation of CPB, resulting in clinically relevant hemodilution [52]. While the small shifts in HCT that predictably occur with the initiation of CPB have been postulated to have a beneficial impact on overall macro- and micro-circulation through reduced blood viscosity, the impact of critically low HCT (<22–24%) has been associated with adverse outcomes such as impaired hemostasis, and poor end-organ perfusion leading to cognitive dysfunction, renal impairment, and gut ischemia [52,53,54]. Additionally, this raises the risk of allogenic blood transfusion. Accordingly, nearly 50% of all cardiac surgery patients receive a transfusion of red blood cells, which increases perioperative morbidity and mortality, and further contributes to the significant amount of blood product consumption by cardiac surgery in general worldwide [53].

Retrograde autologous priming (RAP) is a coordinated effort between the anesthesiologist and perfusionist used prior to the initiation of cardiopulmonary bypass (CPB) to reduce hemodilution associated with initiating bypass [52]. It aims to replace the crystalloid used to prime the circuit with the patient’s own blood, via passive exsanguination from the patient’s arterial and venous cannulation sites [52]. This practice can lead to transient hypotension resulting in decreased perfusion prior to initiation of CPB, eliciting end-organ damage [55], as the preload is sacrificed to prime the circuit. This necessitates concomitant support with vasopressors such as phenylephrine or norepinephrine to ensure hemodynamic stability.

## 6. Anesthetic Considerations

### 6.1. Intraoperative Monitoring

This procedure is performed under general endotracheal anesthesia in a cardiac operating room using cardiopulmonary bypass and transesophageal echocardiography (TEE). Standard American Society of Anesthesiologists (ASA) monitors are applied, and blood pressure is monitored via an intra-arterial catheter, typically in the radial position of the non-dominant hand. While infrequent, if it is anticipated that circulatory arrest will be required to assist in the repair of the ascending aorta or arch, we choose to place a right radial arterial line to monitor antegrade cerebral perfusion.

Temperature monitoring is conducted at two locations using both bladder or rectal and nasopharyngeal temperature probes—with care to properly lubricate and place prior to heparization. Prior to induction, a Bispectral Index (BIS) monitor is placed over the patient’s forehead to monitor anesthetic depth throughout the case. Furthermore, although not used for all our cardiac procedures, our Ross protocol calls for Near-Infrared Spectroscopy (NIRS) cerebral oximetry monitoring due to the surgical complexity and proximity to the aortic arch. However, peripheral intravenous (IV) access alone is sufficient for the induction of anesthesia. Central access is typically obtained post-induction with a 9Fr introducer catheter or Multiaccess Catheter (MAC) in order to monitor central venous pressure (CVP) and for medication administration. External defibrillation pads are placed prior to induction; however, at some institutions, this is reserved for those with stenotic valve lesions or pre-existing coronary artery disease. We do not routinely place pulmonary artery catheters in these patients, as the location of the catheter may obscure the surgical field and sit upon freshly created anastomotic sites.

### 6.2. Anesthesia and Analgesia

General anesthesia is maintained with volatile anesthetics and analgesia with IV opioid narcotics, most commonly fentanyl and/or hydromorphone at our practice. While induction of anesthesia is well tolerated, the use of ANH and RAP necessitates the use of vasopressors and fluid boluses to ensure hemodynamic stability prior to initiation of CPB.

While general anesthesia for the Ross procedure may be maintained with either volatile agents or total intravenous anesthesia (TIVA), current evidence does not demonstrate a definitive advantage of one technique over the other. Volatile anesthetics such as sevoflurane and desflurane have been associated with theoretical cardioprotective effects via ischemic preconditioning pathways, including reduced myocardial biomarker release and shorter hospital stays in some studies [56]. However, large multicenter trials have failed to show a significant difference in major outcomes such as 1-year mortality [57]. As such, no consensus or guideline currently favors volatile anesthesia over TIVA, and the choice remains guided by institutional resources, perfusion equipment capabilities, and provider preference. The perfusion equipment at our institution does not have the capability of providing volatile anesthetics during bypass.

Regarding intraoperative analgesia, there has been emerging data that methadone may offer a safe and effective alternative to traditional intraoperative opioids. Multiple studies have demonstrated the utility of intraoperative IV methadone (with a dose ranging from 0.1 to 0.4 mg/kg, prior to sternotomy) in cardiac surgical patients. As more practices shift towards analgesia optimization via a multimodal approach, methadone may be a valuable tool to reduce pain scores and reduce intraoperative and postoperative opioid requirements [58,59,60,61].

Moreover, ketamine’s role in the cardiac operating room has been examined with similar opioid-reduction goals in mind. Though its use is theoretically appealing, it has had conflicting evidence over the last 10 years. Existing studies have variable dosing regiments from 0.25 mg/kg to 2 mg/kg, but most studies seem to implement a subanesthetic dose ranging from 0.25 mg/kg to 0.5 mg/kg IV bolus. A Cochrane review conducted in 2018 of 130 studies revealed that perioperative intravenous ketamine probably reduces postoperative analgesic consumption and pain intensity [62]. However, its use must be weighed against its potential harm of inducing negative experiences such as hallucinations in the immediate postoperative period [63]. Moreover, it commonly causes tachycardia, which can be detrimental in patients with existing coronary artery disease, stenotic heart lesions, and aortic aneurysms, which require proper impulse control [64]. Our practice selectively incorporates methadone and ketamine into patients’ intraoperative cardiac surgery analgesia plans based on factors like substance-use history and opioid tolerance.

Lastly, there is mounting evidence regarding the safety, analgesic efficacy, and potential reduction of acute kidney injury, regarding the use of intraoperative acetaminophen. We typically employ non-narcotic analgesia with 1 g of IV acetaminophen after sternal closure [65,66]. At the summation of the procedure, the patient remains sedated on infusions of propofol and/or dexmedetomidine in the intensive care unit (ICU).

### 6.3. Regional Anesthesia

Emerging regional anesthetic techniques have also been examined for their safety and efficacy in reducing postoperative pain scores in cardiac surgery. Some evidence suggests the use of an erector spinae plane (ESP) block and catheter for acute postoperative cardiac surgery pain may provide superior analgesia compared to conventional intravenous management [67]. Parasternal nerve blocks, such as the transversus thoracic muscle plane block (TTMPB) as well as the Pecto-Intercostal Fascial Plane Block (PFIB), are both of interest, however, have ultimately failed to show any benefit in terms of length of stay and ICU course [68,69,70,71]. We attribute this largely to the failure of these blocks to adequately cover chest tube and drain insertion sites, rather than sternotomy. Thus, further evidence is needed before these are routinely implemented at our institution.

### 6.4. Separation from Cardiopulmonary Bypass and Hemodynamic Control

Separation from CPB requires cautious hemodynamic optimization but can typically occur in a rapid and safe manner—assuming adequate myocardial protection and surgical hemostasis. While there should be a full TEE examination, a rapid and focused exam of key anatomic structures in the brief period prior to CPB separation is required. This includes evaluation of the autograft for insufficiency greater than trace-mild, the integrity of the homograft, left ventricular (LV) dysfunction, right ventricular (RV) dysfunction, and regional wall motion abnormalities (RWMA).

To facilitate the surgery, the native coronary buttons are removed and implanted into the neo-AV [37]. Therefore, new or worsening LV function may develop due to impaired blood flow to the geographic distribution of the left main coronary artery. Additionally, manipulation of the right ventricular outflow tract (RVOT), implantation of oversized homograft, and excision of the infundibulum can culminate in RV dysfunction [37].

Magnesium sulfate is administered as a 2 g IV bolus after cross-clamp removal to promote the return of normal sinus rhythm. Magnesium offers several benefits when administered to patients undergoing CPB. Specifically, it helps to mitigate the sudden generation of oxygen-derived free radicals associated with calcium administration, and blocks reperfusion-induced free radical formation, thus reducing myocardial insult [72]. In doing so, there is a decreased incidence of postoperative dysrhythmia and ischemia-reperfusion injury, with a magnitude of effect comparable with that of antiarrhythmic drugs [73].

Hypertension is commonly encountered upon separation from CPB during the Ross procedure. This is particularly evident once the preload has normalized (with reinstitution of ANH) and sinus rhythm is present. A tailored surgical approach, combined with tight control of systolic blood pressure and heart rate, appears to mitigate clinically significant early dilatation of the autograft root [74]. We utilize antihypertensive medications such as nicardipine or labetalol, if necessary, to decrease systolic blood pressure to a range of 90 mmHg to 110 mmHg and a heart rate of 60 to 80 beats per minute. Ultimately, patients tolerate this well and leave the hospital with at least one antihypertensive for their first 6 to 12 months postoperatively.

### 6.5. Early Extubation and Fast-Track Recovery After Cardiac Surgery

In the acute postoperative phase following cardiac surgery, early extubation has emerged as a pivotal component of enhanced recovery after surgery (ERAS) and fast-track protocols, especially in younger and healthier patients characteristic of this population. A growing body of evidence supports early extubation—within 6 h—as both safe and effective in appropriately selected cardiac surgical patients.

A single-center prospective study demonstrated that high compliance with an ERAS protocol was associated with significantly reduced time to extubation (median reduction of 3.4 h), increased rates of intraoperative extubation (odds ratio 35.8), and shortened hospital length of stay (median reduction of 19.4 h) without an increase in reintubation rates [75]. Similarly, a Cochrane systematic review of 28 randomized controlled trials showed that fast-track interventions such as low-dose opioid anesthesia and time-directed extubation protocols reduced time to extubation by up to 10.5 h and decreased ICU stay by up to 10.5 h, albeit without affecting the total hospital length of stay [76].

Within our institutional practice, while most cardiac patients are extubated later in the day in the cardiothoracic ICU, this trend aligns with a broader paradigm shift toward early extubation to reduce ICU resource utilization and enhance recovery. These findings underscore the importance of integrating structured fast-track protocols into postoperative care for Ross procedure patients, balancing the benefits of early extubation with individualized risk stratification.

## 7. Intraoperative Transesophageal Echocardiographic Assessment

Patients scheduled for the Ross procedure undergo a gauntlet of preoperative tests and imaging modalities as delineated in prior sections. The role of intraoperative TEE is to confirm these preprocedural features. Postoperatively, its use is to detect the normal functioning of both the autograft, homograft, and ventricular function [29]. As such, TEE is treated as an additional monitor with a diagnostic role in the pre-CPB and post-CPB periods.

### 7.1. Pre-Cardiopulmonary Bypass TEE Assessment

Pre-bypass TEE assessment of the heart for the Ross procedure involves a full echocardiographic assessment of the heart. However, special emphasis is paid to identifying five general components, broken down as follows:

1. Inspection of the native AV
2. Inspection of the native PV
3. Evaluation of LV systolic function and RWMA
4. Evaluation of RV systolic function
5. Evaluation of the ascending aorta and aortic arch

Echocardiographic evaluation of the AV should confirm the structure of the AV (i.e., unicuspid, bicuspid, and tricuspid) as well as the size of the LVOT, AV annulus, aortic root, Sino-tubular junction, and ascending aorta. In patients with aortic stenosis, the aortic and pulmonary annuli are typically very closely matched in size (<2 mm variation). However, in aortic insufficiency, there is a higher risk of geometric mismatch, in which the existing AV annulus is larger than the PV annulus. While this does not preclude the surgery, it does often require further surgical intervention with an extra-aortic annuloplasty [37]. Conversely, if the aortic annulus is smaller than the pulmonary annulus by 2–3 mm, the surgeon may be required to perform a concomitant aortic root enlargement [37].

Given the anterior location of the PV and obscure insonation angles, its assessment can prove challenging by TEE. It is our practice to measure the PV and main pulmonary artery and assess gradients in multiple views including the Upper-Esophageal Aortic Arch Short Axis (UE-Ao Arch SAX), Mid-Esophageal RV Inflow/Outflow (ME-RV Inflow/Outflow), as well as the Mid-Esophageal ascending aorta short and long axis views (ME-Asc Ao SAX and LAX). In general, the ME-RV Inflow/Outflow view displays the PV on the long axis and is useful for detecting pulmonic regurgitation by CFD. The main PA (mPA) and the PV can be seen in the UE-Ao Arch SAX view on the left side of the display by turning the probe until these structures come into view [77]. Retroflexing the probe will often improve the view of the PV. The UE-Ao Arch SAX view usually allows the Doppler beam to be aligned parallel to flow through the PV and main PA and is therefore useful for measuring blood flow velocities through these structures [77]. However, this view can be difficult to obtain in most patients, due to displacement of the TEE probe by a dilated ascending aorta, or the lungs themselves. Therefore, modifications of the Deep Transgastric (DTG) views of the RV and Inflow/Outflow tract can prove useful.

Primary pulmonic regurgitation (PR) is rare when compared to secondary PR. Lesions of the PV leaflets are more common in congenital heart disease [78]. Thus, the role of TEE is to identify and quantify any clinically significant regurgitation or fenestrations in the PV. As it currently stands, there is scant data regarding the quantification of PR. This stems partly from the difficulty in visualizing the PV, the fact that minor degrees of PR are common and have no clinical impact, and the low incidence of clinically significant PR in adults. Therefore, many of the concepts for quantifying PR are derived from the assessment of AI [78].

**Figure 1:**
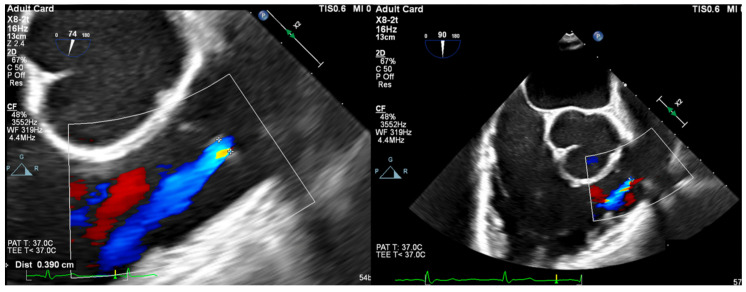
ME RV Inflow/Outflow view with CFD over PV depicting PR jet. The image is frozen and the zoom function allows for measurement of PR vena contracta.

**Figure 2:**
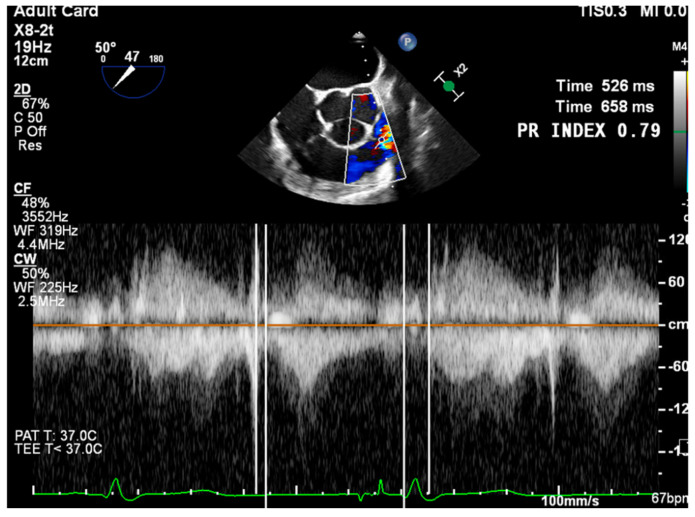
ME RV Inflow/Outflow with continuous wave Doppler (CWD) and angle correct, capturing the spectral Doppler profile of the PR jet. PR duration is measured from its onset in early diastole to the end of the regurgitant Doppler signal. The total diastolic time is measured from the end of the forward pulmonary flow (coinciding with the onset of the retrograde PR flow) to the beginning of the next forward pulmonary flow curve.

**Figure 3:**
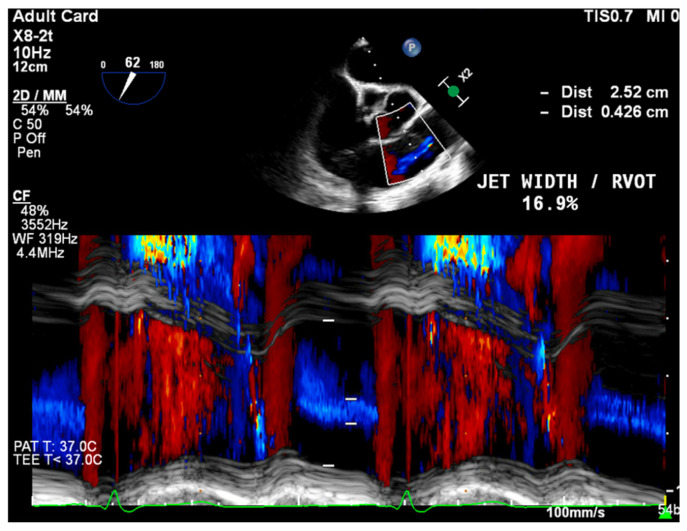
ME RV Inflow/Outflow with M-Mode through the RVOT, capturing both RVOT width and PR jet width.

1. Vena Contracta Width (VCW): Typically not used for multiple jets. Cutoffs for various grades of PR have not been validated, but most use AI cutoffs (Figure 1).
  - VCW/PV ratio has been used with a value of >0.5 consistent with severe PR.
2. PWD flow reversal in branched PA: Affected by PA compliance, so brief flow reversals are normal.
3. CWD: Through the mPA, watch for envelope density. Dense is consistent with moderate to severe PR, faint is more likely to be mild.
4. PR Index: The ratio of PR duration and total diastolic time (regurgitation time/diastole); <0.77 is consistent with severe PR (Figure 2).
5. PR Jet Width/RVOT Width: With >65% consistent with severe PR (Figure 3).

Assessment of LV systolic function should be routinely assessed using 2D and/or 3D calculations, as recommended by the American Society of Echocardiography (ASE). While techniques may vary from physician to physician, standard practice is to apply the Biplane Simpson’s technique using 2D images of the ME-4C and ME-2C. Additionally, utilization of LV full volume analysis with 4–6 beat acquisition, allows for 3D LVEF quantification.

**Figure 4:**
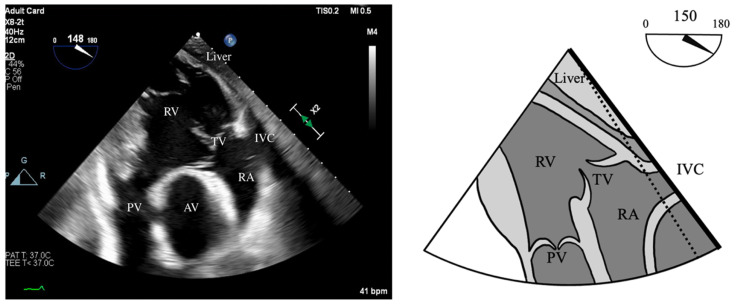
DTG RV Inflow/Outflow view. Acquired in DTG view between 120–150° omniplane, anteflexion, and turned right to center the RV on screen. Allows for alignment of both the lateral tricuspid valve annulus and RVOT.

**Figure 5:**
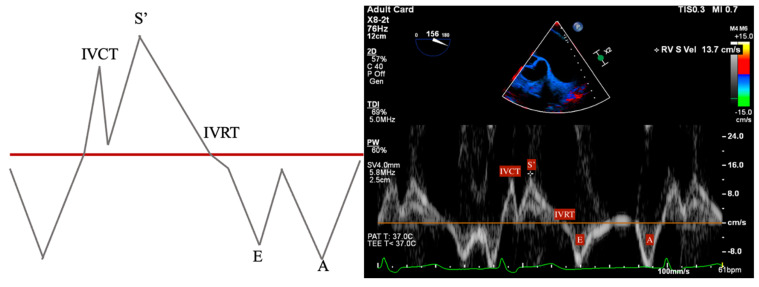
Tissue Doppler Imaging (TDI) of lateral tricuspid valve annulus in DTG RV Inflow/Outflow view. Isovolumetric contraction time (IVCT) and isovolumetric relaxation time (IVRT).

**Figure 6:**
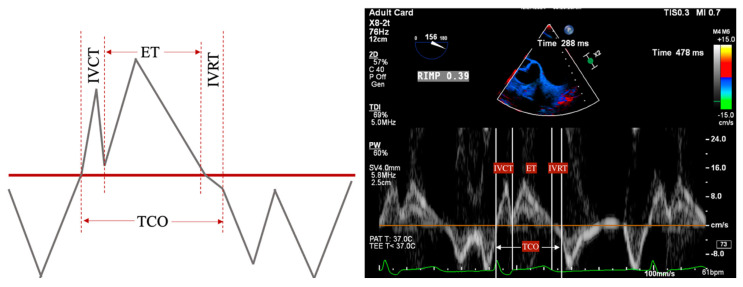
RIMP measurement performed in the DTG RV Inflow/Outflow view. Isovolumetric contraction time (IVCT), isovolumetric relaxation time (IVRT), ejection time (ET), and total contraction time (TCO).

RV function is evaluated both qualitatively and quantitatively. Qualitative measures include the following:

1. RV Fractional Area Change (FAC): Measured in the ME-4C view, normal >35%.
2. Tricuspid Annular Plane Systolic Excursion (TAPSE): ME-RV Inflow/Outflow with angle correction over lateral tricuspid annulus, normal >1.6 cm/s.
3. S’: Measured from a non-standard TEE view. In the Deep Transgastric long axis (DTG-LAX) view, omniplane to 120–150° and turn right. The RV and lateral tricuspid annulus will be centered on the screen. Tissue Doppler Imaging (TDI) is then used to measure the systolic velocity of the lateral tricuspid valve annulus (S’) with >10 cm/s considered normal [79] (Figure 4 and Figure 5).
4. RV Index of Myocardial Performance (RIMP): Similar to S’, total contraction time (TCO) is subtracted from the ejection time (ET) and divided by TCO. The normal value by TDI is <0.54 (Figure 4 and Figure 6).

### 7.2. Post-Cardiopulmonary Bypass TEE Assessment

**Figure 7:**
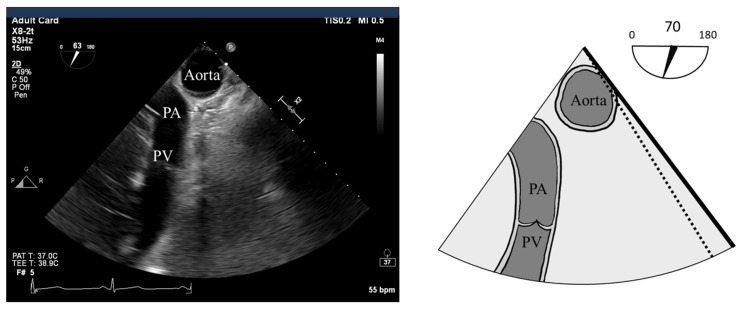
UE AA SAX view: This is typically acquired between 70–90° omniplane, demonstrating the aortic arch in the short axis orientation; the innominate artery may be visualized. Pulmonary artery (PA) and pulmonic valve (PV) are also visualized.

Post-bypass TEE assessment of the heart plays a vital role in assessing the technical adequacy of the Ross procedure. The assessment follows similarly to our pre-bypass exam broken down as follows:

1. Inspection of the neo-AV
2. Inspection of the homograft PV
3. Evaluation of LV systolic function and RWMA
4. Evaluation of RV systolic function
5. Evaluation of the ascending aorta and aortic arch (Figure 7).

Of note, a novel technique to evaluate the integrity of the autograft involves pressurizing the aortic root using the aortic root vent, without the removal of the aortic cross-clamp. This allows for the generation of a pressure gradient between the aortic root and the LV cavity. Echocardiographic evaluation at this point will be difficult due to the lack of LV filling; however, torrential issues within the autograft can be identified and therefore fixed before re-warming.

## 8. Conclusions

The Ross procedure represents a unique and valuable option for aortic valve replacement, particularly in younger patients seeking to avoid lifelong anticoagulation and prosthetic valve-related complications. Despite its technical complexity and decline in utilization, the Ross procedure has demonstrated favorable long-term outcomes in select patient populations. Successful outcomes rely heavily on a comprehensive preoperative assessment, meticulous intraoperative technique, and tailored anesthetic management that addresses the procedure’s unique considerations. Intraoperative transesophageal echocardiography plays a crucial role in guiding surgical decision-making and assessing the adequacy of the autograft and homograft. Blood conservation techniques such as acute normovolemic hemodilution and retrograde autologous priming can mitigate hemodilution and transfusion requirements associated with cardiopulmonary bypass. Anesthetic management focuses on optimizing hemodynamics, myocardial protection, and pain control while minimizing complications. As the Ross procedure continues to evolve and find its place in the armamentarium of aortic valve interventions, ongoing research and refinement of surgical and anesthetic techniques will be instrumental in further improving patient outcomes. A multidisciplinary approach, encompassing the expertise of cardiac surgeons, anesthesiologists, and allied specialties, remains paramount in delivering optimal care for patients undergoing this transformative procedure.
